# Effects of losartan in patients with NAFLD: A meta-analysis of randomized controlled trial

**DOI:** 10.1515/biol-2022-0583

**Published:** 2023-03-21

**Authors:** Chang Meng, Zejun Song, Lingnan Zhang, Yu Geng, Jing Sun, Guobin Miao, Peng Liu

**Affiliations:** Department of Emergency, Emergency General Hospital, XiBaHe South Road 29, Chaoyang District, Beijing, 100028, PR China; Department of Gastroenterology, Beijing Tsinghua Changgung Hospital, School of Clinical Medicine, Tsinghua University, No. 168 Litang Road, Changping District, Beijing, 102218, PR China; Department of Cardiology, Affiliated Hospital of Hebei University, Hebei University, 212 Yuhua East Road, Lianchi District, Baoding City, 071000, PR China; Department of Cardiology, Beijing Tsinghua Changgung Hospital, School of Clinical Medicine, Tsinghua University, NO. 168 Litang Road, Changping District, Beijing 102218, P. R. China; Department of Critical Care Medicine, Emergency General Hospital, XiBaHe South Road 29, Chaoyang District, Beijing, 100028, PR China; Department of Cardiology, Ordos Central Hospital, Ordos School of Clinical Medicine, Inner Mongolia Medical University, 23 Yijinhuoluo West Street, Dongsheng District, Inner Mongolia, 017000, PR China

**Keywords:** losartan, liver function, blood lipids, non-alcoholic fatty liver disease

## Abstract

Losartan has become a hot spot in the treatment of non-alcoholic fatty liver disease (NAFLD) among angiotensin receptor blocker drugs. We sought to conduct a systematic examination and meta-analysis to examine the effects of losartan on patients with NAFLD. We searched for potentially randomized controlled trials in PubMed, Embase, China National Knowledge Infrastructure, Wanfang, and the Cochrane database up to October 09, 2022. We used the Cochrane risk of bias tool to evaluate the study quality. Analysis of subgroups, sensitivity analysis, and publishing bias were explored. The quality of the included studies was moderate to high. Six trials involving 408 patients were included. The meta-analysis demonstrated that aspartate transaminase was significantly affected by losartan therapy (mean difference [MD] = −5.34, 95% confidence interval [CI] [−6.54, −4.13], *Z* = 8.70, *P* < 0.01). The meta-analysis subgroup showed that losartan 50 mg once daily could lower the level of alanine aminotransferase (MD = −18.92, 95% CI [−21.18, −16.66], *Z* = 16.41, *P* < 0.01). There was no statistically significant difference in serum total cholesterol, triglyceride, low-density lipoprotein, and high-density lipoprotein.

## 1 Introduction

Non-alcoholic fatty liver disease (NAFLD) is a progressive disease characterized by excessive accumulation of fat in the liver, typically characterized by simple steatosis at the onset. These include non-alcoholic steatohepatitis (NASH), liver fibrosis, cirrhosis, and hepatocellular carcinoma [1]. Currently, the pathogenesis of this disease is believed to be linked to obesity, insulin resistance, type 2 diabetes mellitus, and hyperlipidemia. In recent years, with the growing incidence of obesity and diabetes, NAFLD has become the most common chronic liver disease, with about 25% of the global population suffering from NAFLD [2]. In addition to lifestyle interventions, such as exercise and diet, there is still a lack of specific drugs to treat NAFLD [3]. The pathophysiology of NAFLD, particularly involving insulin resistance and subclinical inflammation, is closely related not only to these non-communicable diseases but also to the severe course of the infectious disease COVID-19. Damage to glucose and lipid metabolic pathways, driven by the global rise in obesity and type 2 diabetes, is likely to be behind the increase in NAFLD patients [4]. Some studies have also found that the pathophysiological mechanism of NAFLD is closely related to liver and fat metabolism [5]. It is known that alanine aminotransferase (ALT) and aspartate aminotransferase (AST) are indicators of the degree of hepatocyte damage. A few studies [11,15,16] have observed that losartan may play a role in reducing transaminase in NAFLD population.

In some studies, angiotensin receptor blockers (ARBs) have been found to regulate hepatic lipid [4]. In the absence of AT1R, lipid accumulation is reduced and PPAR is significantly induced. Therefore, AT1R blockade may be effective in the treatment of NAFLD or NASH [6]. Although the function of ARBs has been widely used in animal experiments to prevent NAFLD complications, there is a lack of clinical data on patients [7], and the effectiveness of ARBs in the treatment of NAFLD is still controversial. For example, the effects of losartan on biochemical variables, hepatic steatosis, inflammation, and serum biomarkers of fibrosis in patients have also been mixed [8,9].

Thus, reported results have been contradictory. Among all ARBs, losartan has the largest number of randomized controlled clinical studies on NAFLD. In this study, we carried out a meta-analysis to investigate the effects of losartan on liver function and blood lipids in patients with NAFLD.

## 2 Methods

Our protocol has been registered on the International Platform of Registered Systematic Review and Meta-analysis Protocols database (registration number: INPLASY2022110006, DOI number: 10.37766/inplasy2022.11.0006).

### 2.1 Search strategy

Two independent researchers (CM and ZJS) conducted extensive electronic searches for relevant articles published as of October 9, 2022. The database includes PubMed, Embase, China National Knowledge Infrastructure, Wanfang, and the Cochrane database. English retrieval uses the medical subject title (MeSH) in combination with the following terms to search: “nonalcoholic fatty liver disease” and “losartan.” Manually select relevant randomized controlled trials (RCTs). The Chinese search uses subject words or synonyms, including “nonalcoholic fatty liver disease,” “losartan,” and “ARB.”

### 2.2 Literature screening and data extraction

EndNote (X9 version) software is selected for document management; two investigators independently evaluated the eligibility of the identified items. The title and summary are filtered for the first time, and qualified articles are reserved for full-text review. Inclusion criteria for studies meeting the following requirements include (1) adults or children clinically diagnosed as NAFLD; (2) treatment with losartan; and (3) outcomes’ indicators, ALT, AST, Tc, Tg, high-density lipoprotein (HDL), and low-density lipoprotein (LDL), including one. The subject may be included in the study. The exclusion criteria are as follows: (1) non-human research; (2) non-randomized controlled trial; (3) there are not enough data to extract, such as the summary of some meetings; literature materials such as review and pharmacological introduction; and (4) animal experiment.

### 2.3 Bias and quality assessment

The two researchers independently evaluated, preliminarily selected and checked the literature data according to the unified and standardized method, included them in the literature in strict accordance with the admission and exclusion criteria, then collected information, and evaluated the quality of selected articles according to the quality evaluation standard of Cochrane Reviewer Handbook 5.1.0 [10] (random sequence generation, allocation concealment, blinding of participants and personnel, blinding of outcome assessment, incomplete outcome data, selective reporting, and other bias).

### 2.4 Statistical analysis

Revman5.3 software is used for meta-analysis. Data that meet homogeneity (*P* > 0.10 and *I* ^2^ ≤ 50%) through the heterogeneity test are meta-analyzed with the fixed-effect model. If homogeneity (*P* ≤ 0.10 or *I* ^2^ > 50%) is not met, and heterogeneity cannot be ruled out, random-effect model can be used to combine effects, but it should be noted that sensitivity analysis and subgroup analysis should be considered for the type of analysis data. The mean difference (MD) and 95% confidence interval (CI) were calculated.

## 3 Results

**Figure 1:**
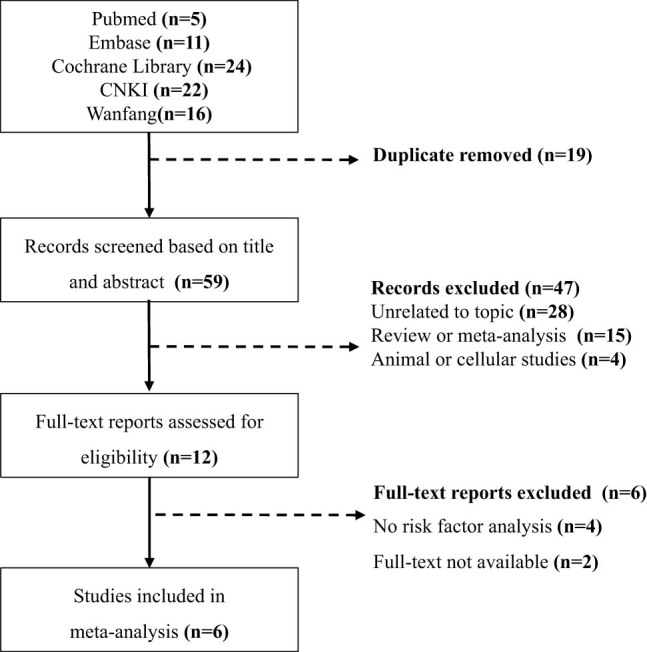
Selection process for studies included in the meta-analysis.

According to the literature search results, 78 literatures were initially retrieved, 19 duplicates were removed, 47 literatures such as review and irrelevant topics were excluded from reading titles and abstracts, 6 literatures that did not meet the inclusion criteria were excluded from reading the full text, and 6 literatures were finally included, including 4 [11,12,13,14] in English and 2 [15,16] in Chinese. The detailed process is shown in Figure 1.

The basic characteristics of literature and the quality evaluation of methodology included 6 articles and 408 researchers in total. The baseline characteristics included in the study are shown in Table 1. There was no statistical difference in the baseline characteristics of all patients, and the quality of the included literature was moderate to high. The quality evaluation of the literature are shown in the supplement.

**Table 1:** Summary of the included studies

Study	Year	Study design	*N*	Included patients	Intervention	Control	Findings	Follow up
McPherson et al. [11]	2017	P,MC	32	Age > 18; NAFLD	Losartan 50 mg qd	Placebo	ALT, AST, TC, TG, LDL, HDL, FS	96 weeks
Vos et al. [12]	2022	P,MC	83	Age 8–17 years; NAFLD	Losartan 100 mg qd	Placebo	ALT, AST	24 weeks
Fogari et al. [13]	2012	P,SC	150	Age > 18; NAFLD	Losartan 100 mg qd	Amlodipine 10 mg/day	ALT, AST, TC, TG, LDL, HDL	12 months
Hirata et al. [14]	2013	P,SC	17	Age > 20; NAFLD	Losartan 100 mg qd	Telmisartan 20 mg once daily	ALT, AST, TC, TG, HDL	12 months
Chen [15]	2020	P,SC	76	Age > 18; NAFLD	Losartan 50 mg qd	Conventional therapy	ALT, AST	6 months
Liu et al. [16]	2019	R,SC	50	Age > 18; NAFLD	Losartan 50 mg qd	Placebo	ALT, AST, TC, TG, FS	96 weeks

P, prospective; R, retrospective; MC, multicenter; SC, single center; AST, aspartate aminotransferase; ALT, alanine aminotransferase; NAS, non-alcoholic fatty liver disease; FS, fibrosis stage; qd, once daily.

**Figure 2:**
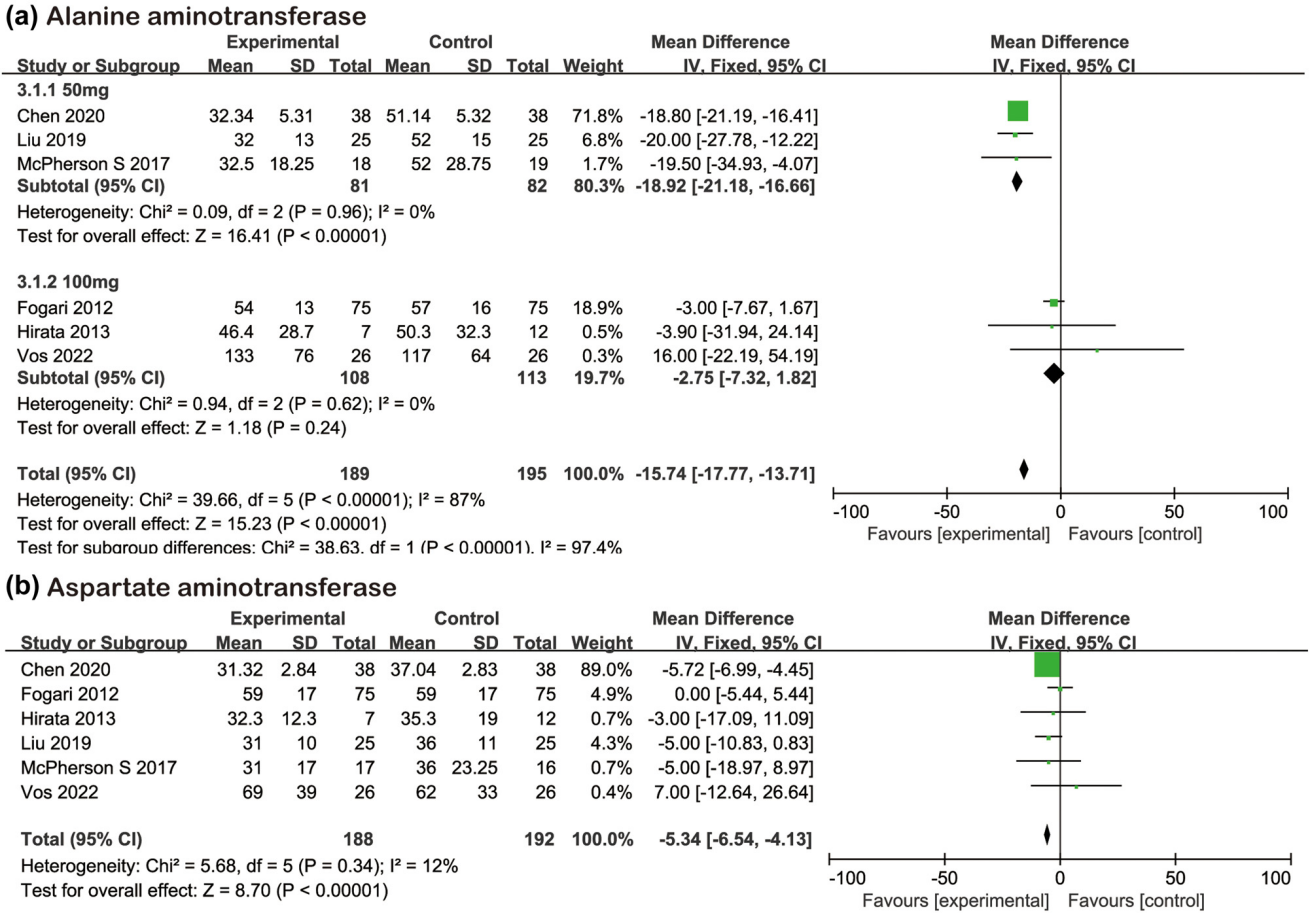
Forest plots of the losartan therapy on liver function. (a) ALT and (b) aspartate aminotransferase.

This study mainly discussed the effect of losartan on liver function and blood lipid in nonalcoholic patients. Six studies [11,12,13,14,15,16] reported the change of ALT, and there was heterogeneity between literatures. Random effects were used for analysis. Meta grouping results showed that losartan could reduce the ALT level of NAFLD, MD = −15.74, 95% CI [−17.77, −13.71], *Z* = 15.23, *P* < 0.01. Based on statistics and clinical analysis, we further used sensitivity analysis and subgroup study to group the patients according to the dosage of losartan. We found that the subgroups could be analyzed according to the fixed effect (*I* ^2^ = 0%, *P* > 0.10). The meta-analysis subgroup results showed that losartan 50 mg once daily [11,15,16] could reduce the ALT level (MD = −18.92, 95% CI [−21.18, −16.66], *Z* = 16.41, *P* < 0.01). The losartan 100 mg once daily [12,13,14] group failed to show statistical difference between the experimental group and the control group and only showed a downward trend (MD = −2.75, 95% CI [−7.32, 1.82], *Z* = 1.18, *P* = 0.24). The detailed process is shown in Figure 2a. Six studies [11,12,13,14,15,16] reported the changes of AST (Figure 2b). There was no heterogeneity between the literatures. Fixed effects were used for analysis. Meta-analysis showed that losartan could reduce the AST level in the experimental group (MD = −5.34, 95% CI [−6.54, −4.13], *Z* = 8.70, *P* < 0.01).

**Figure 3:**
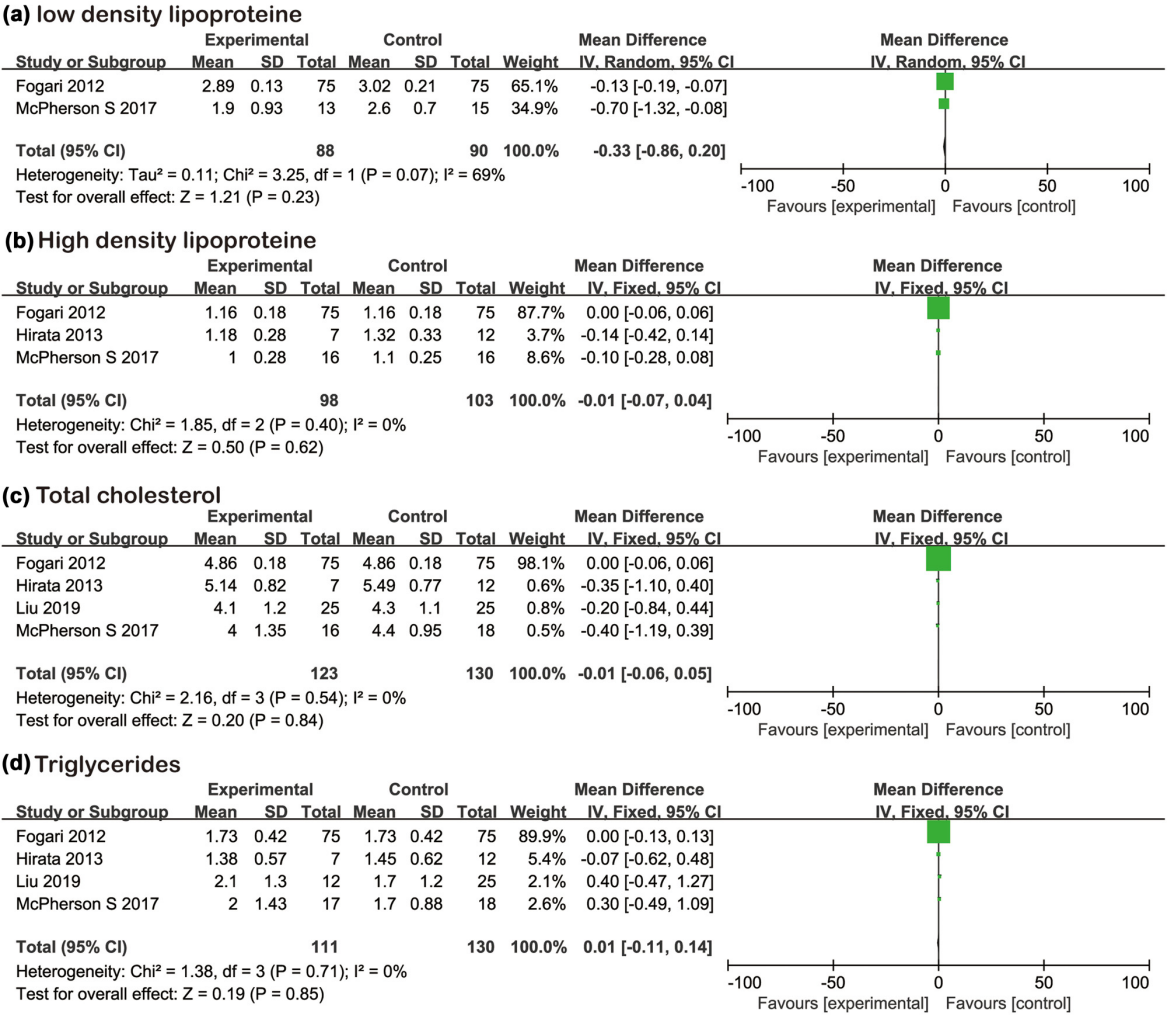
Forest plots of the losartan therapy on blood lipids. (a) LDL; (b) HDL; (c) total cholesterol; and (d) triglyceride.

Two studies [11,13] reported the changes in LDL (Figure 3a), and there was heterogeneity between literatures. Randomized effects were used for analysis. Meta-analysis showed that losartan did not show statistical difference between the experimental group and the control group (MD = −0.33, 95% CI [−0.86,0.20], *Z* = 1.21, *P* = 0.23). Three studies [11,13,14] reported the changes in HDL (Figure 3b). There was no heterogeneity between the literatures. Fixed effects were used for analysis. Meta-analysis showed that losartan did not show statistical difference between the experimental group and the control group (MD = −0.01, 95% CI [−0.07,0.04], *Z* = 0.50, *P* = 0.62). Four studies [11,13,14,16] reported the changes in TC (Figure 3c) and TG (Figure 3d). There was no heterogeneity between the literatures. Fixed effects were used for analysis. Meta-analysis showed that losartan in the experimental group did not show statistical difference between the experimental group and the control group (MD = −0.01, 95% CI [−0.06,0.05], *Z* = 0.20, *P* = 0.84) (MD = 0.01,95% CI [−0.11,0.14], *Z* = 0.19, *P* = 0.85).

The funnel chart analysis (publication bias analysis) of the indicators included in the study shows that the inverted funnel chart of each indicator is basically symmetrical, indicating that there is no publication bias. However, the number of relevant studies is relatively small, and there may be some errors in the analysis of the inverted funnel chart.

## 4 Discussion

At present, NAFLD is the most common but also the most easily ignored disease by patients and clinicians, and its related complications include liver dysfunction, portal hypertension, liver fibrosis, and cirrhosis. However, in clinical practice, NAFLD is still mainly treated by lifestyle interventions, such as diet adjustment and exercise. Currently, the clinical or guidelines are temporarily. There is no drug specifically recommended. Moreira de Macêdo [17] found that the renin–angiotensin system (RAS), as an important regulator, plays an important role in the metabolic process of the body. The potential therapeutic effect of angiotensin-converting enzyme 2 (ACE2) on the regulation of RAS has become a new research direction. Meta-analysis was used in this study to analyze the effect of ACEI/ARB drugs on NAFLD and provide evidence of evidence-based medicine for the clinical application of ACEI/ARB.

The mechanisms of ACEI/ARB in the treatment of NAFLD mainly include the following three aspects: (1) improving insulin resistance (IR). On the one hand, angiotensin II can improve IR by regulating the expression of certain signaling molecules in liver, muscle, and adipose tissue and enhancing insulin negative feedback [18]. Meanwhile, it can reduce systolic blood pressure to a certain extent [19]. (2) ACEI/ARB can improve oxidative stress by upregulating the transcription of ACE2 mRNA; ACEI/ARB can inhibit the c-Jun amino-terminal kinase pathway activated by reactive oxygen species (ROS) by upregulating the expression of ACE2 gene mRNA. At the same time, it can improve local tissue perfusion and oxygenation by reducing Ang Ⅱ-mediated vasoconstriction, reduce the increase of ROS caused by fatty acid accumulation, and inhibit nuclear factor kB. The activation of the pathway can further improve the occurrence of metabolic diseases related to inflammation by blocking the activation of related inflammatory factors [20,21,22]. (3) ARBs can be activated as selective peroxisome proliferators to play a protective role in the liver. At the same time, lipid metabolism can be further improved through the activation and enhancement of adiponectin [23,24]. It can also downregulate the expression of liver sterol regulatory factor-binding protein, inhibit the uptake of lipids by liver cells, reduce hepatocyte steatosis, inhibit the overexpression of cytokine signal transduction inhibitor 3 (SOC-3) in liver tissue, and improve IR and homeostasis of grape [25]. Therefore, ARBs has become a promising new strategy for the prevention and treatment of chronic liver disease, as well as a new therapeutic option for the prevention and treatment of chronic liver disease.

## 5 Conclusion

Meta-analysis results of this study show that losartan can reduce the level of glutamic oxaloacetic transaminase in patients with NAFLD. In the subgroup analysis, losartan 50 mg once a day can reduce the level of glutamic oxaloacetic transaminase, and the clinical effect is more accurate. This study shows that the effect of losartan on improving lipid (TC, TG, LDL) levels is not ideal. The results of this study not only summarize the population of different countries but also creatively include the NAFLD population of the underage [12]. While further increasing the sample size, the results of all populations are also comprehensively presented. To sum up, losartan 50 mg once daily can significantly improve the liver function of NAFLD patients, and its clinical efficacy is relatively accurate. If there is no contraindication, it should be used as early as possible. We can also find that there are few large sample RCTs about losartan in the treatment of NAFLD, and more high-quality RCTs are needed to supplement and evaluate. Of course, this study also has some limitations. The current number of studies is relatively small, and more RCTs are needed to support it in the future.
